# Dietary, Nutrient, and Supramolecular Nanofiber Modulation of the Liver Sinusoidal Clearance System in Metabolic Diseases and Aging

**DOI:** 10.3390/biomedicines14081834

**Published:** 2026-08-14

**Authors:** Binod Pokharel, Anokhi Kulkarni, Rebecca Drager, Fatima Atta Muhammad, Ouliana Ziouzenkova

**Affiliations:** 1 Department of Human Sciences, College of Education and Human Ecology, The Ohio State University, Columbus, OH 43210, USA; pokharel.18@buckeyemail.osu.edu (B.P.); kulkarni.375@buckeyemail.osu.edu (A.K.); attamuhammad.1@buckeyemail.osu.edu (F.A.M.); 2 Department of Biochemistry, College of Arts and Sciences, The Ohio State University, Columbus, OH 43210, USA; drager.4@buckeyemail.osu.edu

**Keywords:** liver sinusoidal clearance system, liver sinusoidal endothelial cells, advanced glycation end products, nanofiber-based therapeutics, fenestration

## Abstract

In modern societies, the renewed concept of food as medicine coexists with unprecedented consumption of highly processed foods, food additives, environmental xenobiotics, and pharmacological agents, contributing to the increasing prevalence of metabolic and degenerative diseases and accelerated aging. Although modern pharmacotherapies have transformed disease management, long-term drug exposure introduces additional metabolic burdens, off-target effects, and cumulative toxicities that are profoundly influenced by nutritional status. Collectively, dietary constituents, environmental chemicals, endogenous metabolic by-products, and therapeutic agents constitute a complex exposome that requires continuous recognition, utilization, detoxification, and clearance. Within this context, the liver sinusoidal clearance system (LSCS) emerges as a central regulator of systemic homeostasis. We propose a conceptual framework in which circulating molecules are classified as self (S), modified self (M), and foreign (F) molecules according to their physiological handling by the LSCS. Through coordinated hepatic utilization of S molecules and selective clearance of M and F molecules, fenestrated liver sinusoidal endothelial cells (LSECs) maintain metabolic homeostasis, immune tolerance, and physiological pharmacokinetics. Conversely, chronic dietary overload, poor dietary quality, food processing, and sustained exposure to pro-inflammatory and oxidative dietary and environmental molecules initiate chronic low-grade inflammation, which promotes LSEC capillarization, impairs hepatic clearance, increases the modification of S molecules into M molecules and establishes a feed-forward cycle that further amplifies chronic inflammation and metabolic dysfunction. Finally, we discuss recent advances in the programmable modulation of the LSCS, including its transient suppression to prolong therapeutic exposure and its activation to enhance the clearance of metabolically harmful M and F molecules through coordinated upregulation of the endoglin–stabilin-2–FcγRIIb axis and the LSEC markers *Oit3* and *Dnase1L3*. We highlight dual-function supramolecular nanofiber platforms that enable bidirectional regulation of the LSCS through nanofiber complexes with therapeutic proteins, thereby expanding their therapeutic potential and enhancing efficacy in the treatment of metabolic, inflammatory, and age-related diseases.

## 1. Introduction

Food is an exceptionally complex matrix, and dietary patterns characteristic of the Western diet are now recognized as major contributors to chronic inflammation, metabolic dysfunction, degenerative diseases, and accelerated aging [1]. Conversely, anti-inflammatory dietary patterns, including the Mediterranean, MIND, and low-salt diets, together with numerous bioactive nutrients, have demonstrated preventive and therapeutic potential and are increasingly recommended as adjunctive strategies for metabolic diseases and cancer [2]. Beyond their nutritional value, food contains a diverse spectrum of endogenous metabolites, xenobiotics, food additives, antibiotics, and naturally occurring phytochemicals of non-human origin [3]. From a homeostatic perspective, all these compounds can be broadly conceptualized as self-like molecules that support normal metabolism and foreign-like molecules that elicit detoxification and clearance pathways. Under metabolic stress or pathological conditions, endogenous self-like molecules may undergo chemical or structural modifications that convert them into foreign-like species, increasing the burden on physiological clearance systems responsible for maintaining metabolic homeostasis [4,5].

Many dietary phytochemicals, including polyphenols, flavonoids, and terpenoids, circulate bound to albumin or lipoproteins and are subsequently cleared by the liver sinusoidal clearance system (LSCS) together with other hydrophobic compounds and modified macromolecules [6]. Likewise, essentially all therapeutic agents, including biologics and gene-based therapies, are recognized as foreign molecules and are therefore subject to hepatic clearance. Depending on the therapeutic modality, from only a few percent to more than 90% of an administered dose may be removed from the circulation before reaching its target tissues, representing a major challenge for achieving effective drug exposure while minimizing off-target toxicity [7]. LSCS provides continuous physiological clearance that preserves metabolic homeostasis while minimizing immune stimulation [8]. Although physiological clearance also relies on renal filtration of small water-soluble metabolites and immune-mediated elimination of foreign material, growing evidence supports a central role for the LSCS in regulating systemic exposure to the dietary and therapeutic exposome [8]. The physiological mechanisms governing LSCS adaptation to nutritional and metabolic cues, their role in metabolic disease progression, and their potential for therapeutic modulation represent major gaps in current knowledge.

In this review, we discuss the mechanisms by which the unique structure of the LSCS enables physiological adaptation to nutritional and metabolic challenges while maintaining metabolic homeostasis and immune quiescence. We then examine how the excessive molecular burden associated with processed diets and sustained exposure to dietary, therapeutic, and environmental molecules progressively challenge the physiological processing and clearance capacity of the LSCS using representative examples discussed throughout this review. We propose that persistent overload of these clearance mechanisms drives LSCS remodeling and functional impairment, establishing a feed-forward cycle that perpetuates chronic inflammation, metabolic dysfunction, and aging. Finally, we discuss emerging nanofiber-based strategies that enable programmable enhancement or suppression of LSCS activity [9], highlighting their potential to improve therapeutic delivery and efficacy of therapeutic protein cargo. These dual-function nanofiber–protein complexes restore physiological clearance while promoting the elimination of metabolic and inflammatory waste, thereby improving therapeutic efficacy in the treatment of chronic inflammatory, metabolic, and age-related diseases.

## 2. Homeostatic Function of the LSCS

From a homeostatic perspective, molecules can be classified into three categories S, M, and F:

**S denotes** dietary and enzymatically produced **self-molecules or their complexes** that are recognized by specialized physiological pathways through structural complementarity to specific receptors and/or transporters and participate in metabolic processes that maintain physiological homeostasis. These molecules include glucose, amino acids, fatty acids, cholesterol, vitamins, bile acids, their enzymatically generated metabolites, and intact complex molecules such as proteins, carbohydrates, and nucleic acids.

**M denotes chemically or structurally modified self-molecules or their complexes** (Table 1) that progressively lose their affinity for specific receptors and other recognition machinery. Chemical modifications include glycation, oxidation, nitrosylation, and other covalent alterations [5,10], whereas modifications of complex molecules and molecular complexes include misfolding, denaturation, electrostatic or hydrophobic interactions with other molecules, and aggregation [11]. Examples include advanced glycation end products (AGEs), minimally modified and oxidized lipoprotein complexes, apoptotic fragments, and other DAMP molecules (Table 1). From the perspective of H-type scavenger receptors of LSCS such as stabilin-1, stabilin-2, FcγRIIb, the mannose receptor, and other components, these modified self-molecules increasingly resemble foreign molecular patterns and become preferential targets for scavenger-mediated clearance.

**F denotes foreign or exogenous molecules or their complexes** that fall outside the body’s normal metabolic and structural networks and are handled predominantly by detoxification and clearance systems—such as scavenger receptors, Toll-like receptors (TLRs), and other PAMP/DAMP-sensing inflammatory pathways—rather than being processed to support metabolic homeostasis. Importantly, some F molecules exhibit incidental or designed structural complementarity to physiological receptors and can modulate metabolic signaling, thereby producing beneficial therapeutic effects or adverse side effects. Examples include pharmacological agents, caffeine, and plant alkaloids, which can produce beneficial therapeutic effects but may also induce adverse side effects.

The self-to-foreign molecular ratio (SFR) is a simple way to describe the balance between molecules that circulate in the body and support metabolism (S, self-molecules) and modified self (M) and foreign (F) molecules that must be removed by the LSCS. This clearance limits their pathological signaling and prevents them from overloading the hepatic clearance system. A high SFR means that most circulating molecules are normal self-molecules. A low SFR means that M and F molecules become more abundant because they are produced faster than the LSCS can remove them or because the LSCS no longer clears them efficiently. The prehepatic SFR (SFR*pre*) describes the molecular composition of blood entering the liver:

$$SFRpre=\frac{S}{M+F}$$

After blood passes through the LSCS, the posthepatic SFR (SFRpost) reflects how effectively the LSCS has removed M and F molecules while maintaining the availability of S molecules during hepatic passage. A healthy, fenestrated LSEC increases the SFR of blood leaving the liver. Fenestrated LSECs preferentially remove M and F molecules through scavenger pathways while facilitating the partial intrahepatic uptake, processing, and redistribution of S molecules, including lipoprotein complexes. The remaining S molecules continue to circulate, ensuring their availability to other tissues. The parameter η represents the clearance efficiency of the LSCS for S, M, and F molecules.

$$SFRpost=\frac{\left(1-\eta s\right)S}{\left(1-\eta M\right)M+\left(1-\eta F\right)F}$$

The **pre**hepatic SFRpre of blood entering the liver is lower than the **post**hepatic SFR (SFRpost) exiting the LSCS, because the LSCS preferentially removes F and M molecules from the circulating pool during each pass through the liver.

The value of η  is likely to differ among S, M, and F molecules because these classes engage distinct specific and scavenger receptors, as well as structurally different pores and fenestrae, to cross the endothelial barrier via transcellular and paracellular pathways [12,17,18]. However, the relative clearance efficacy of these molecular classes has not yet been systematically evaluated under controlled experimental conditions. Physiologically, a major function of LSECs is to maximize LSCS clearance efficacy η, thereby enhancing the selective removal of F and M molecules [19] and limiting transient increases in these inputs during postprandial, neuroendocrine, and immune challenges. As a result, blood leaving the LSCS becomes relatively enriched in native S molecules and depleted of potentially inflammatory or toxic M and F molecules. This supports metabolic homeostasis and immune quiescence [20].

**Figure 1:**
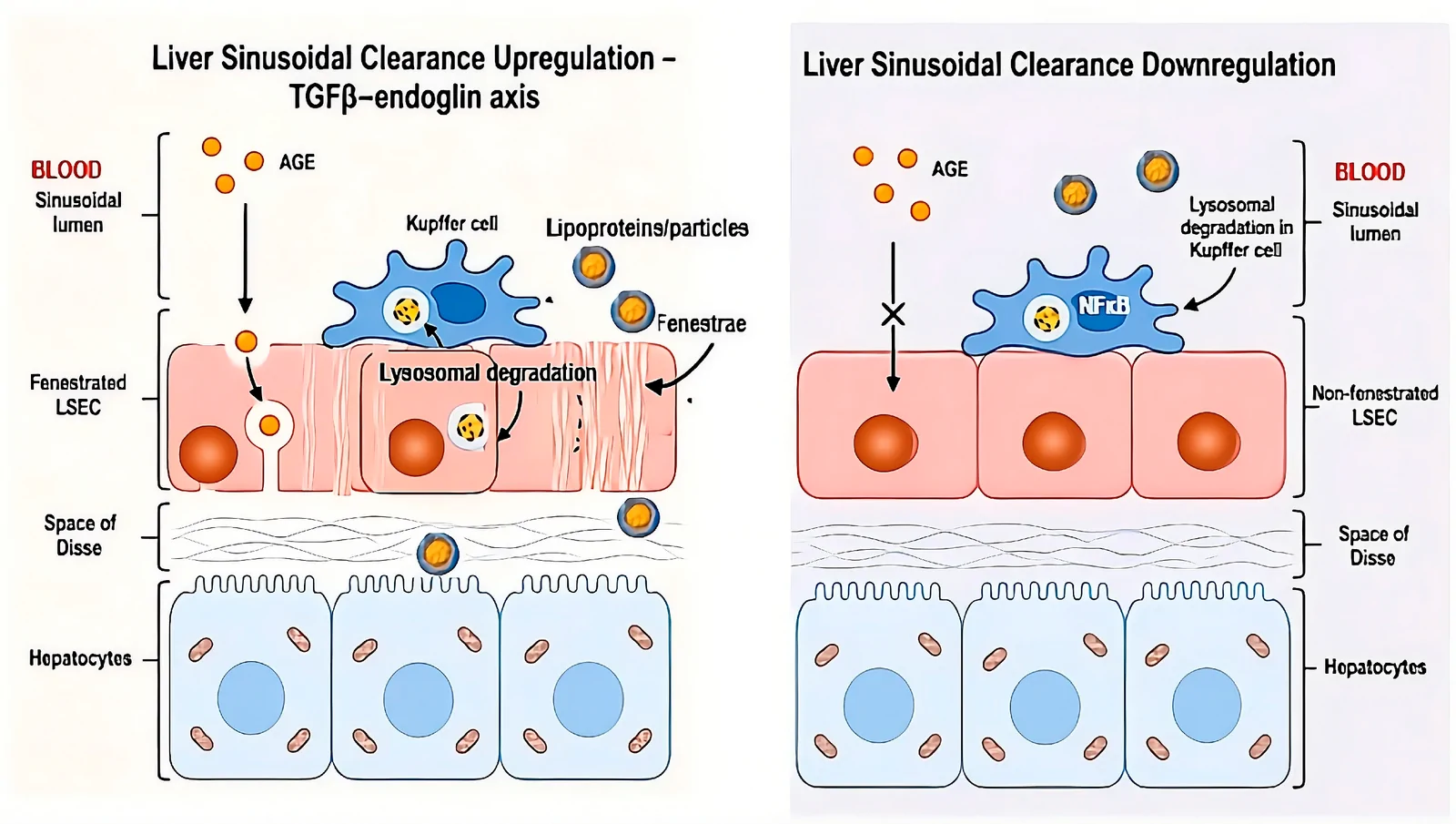
Simplified schematic of the LSCS in fenestrated (**left**) and capillarized (**right**) states of LSEC. In the fenestrated state (**left** panel), LSEC form transcellular fenestrae that span the thin endothelium from the sinusoidal blood lumen to the space of Disse, allowing passage of circulating M molecules, e.g., AGEs-modified proteins, and true F macromolecules. Wavy lines represent extracellular matrix (ECM) deposition in the space of Disse. LSEC exhibit exceptionally high endocytic capacity and, via receptor-mediated endocytosis, actively scavenge, internalize, and degrade these substrates, while macromolecules and complexes, including lipoproteins and chylomicron remnants, traverse LSEC fenestrae and are further metabolized and detoxified by hepatocytes, supporting efficient hepatic clearance of AGEs and related M and F molecules. Each fenestra is surrounded by a fenestra-associated cytoskeletal ring, morphologically described as ~16 nm filaments and now assigned to an actin–spectrin (fodrin) membrane scaffold that regulates fenestra diameter and open/closed state [20]. Proper coupling between caveolin 1 (Cav1), cortical F-actin, and this actin–spectrin scaffold is required to maintain open fenestrae, as disruption of Cav1 or its associated actin remodeling leads to defenestration and capillarization. Kupffer cells also participate in receptor-mediated internalization and phagocytic clearance of pathogen- and damage-associated molecular patterns (PAMPs and DAMPs), for example via TLRs and other pattern-recognition receptors [21]. Engagement of these receptors triggers NF-κB-dependent transcriptional programs, including ROS production and release of pro-inflammatory mediators, thereby sustaining chronic inflammation and promoting defenestration, basement membrane deposition, and capillarization of LSEC. Capillarized LSEC (**right** panel), with loss of fenestrae, formation of a continuous basement membrane, and impaired endocytic function, reduce hepatic access to circulating lipoproteins and chylomicron remnants, thereby limiting sinusoidal clearance capacity (ŋ) and contributing to systemic dyslipidemia (Section 3).

Conversely, when LSECs lose fenestrations or become capillarized (Figure 1), their ability to filter and internalize M and F molecules declines. As a result, the LSCS no longer increases the SFR efficiently during hepatic passage. M and F molecules remain in the circulation longer, increasing the burden on immune-mediated clearance pathways and promoting chronic inflammation. Thus, the SFR provides both a simple conceptual framework and a quantitative measure of LSCS function, illustrating how deterioration of the liver sinusoidal vascular structure reduces hepatic clearance capacity and shifts the circulation toward metabolic and inflammatory dysfunction.

## 3. Structural and Functional Features of LSCS

To distinguish S, M, and F molecules and direct them toward utilization, detoxification, or clearance, the LSCS is organized within the hepatic sinusoidal bed. This structure consists of specialized fenestrated LSEC and Kupffer cells embedded in a reticular, type III collagen-rich extracellular matrix (Figure 1 left panel) [22,23]. The fenestrated phenotype of LSECs facilitates the hepatic influx and utilization of S molecules while promoting the selective clearance of M and F molecules. Kupffer cells, a class of resident macrophages, provide additional scavenging capacity, couple clearance to inflammatory responses, and communicate with innate lymphoid and invariant natural killer T (iNKT) cells [24].

After passing through LSEC fenestrae into the space of Disse by diffusion, receptor-mediated uptake, and endocytosis-linked transcytosis [25], the concentrations of F and M molecules are reduced relative to sinusoidal plasma, whereas S molecules readily equilibrate between these compartments [26]. The space of Disse is a narrow perisinusoidal compartment containing ECM and accommodating hepatocyte microvilli, Kupffer cell processes, and hepatic stellate cells [27]. The narrow perisinusoidal space and its ECM substantially reduce fluid flow relative to the sinusoidal lumen, favoring diffusion-mediated exchange and facilitating communication among hepatocytes, stellate cells, and resident immune cells. Hepatocytes then carry out coordinated phase I and phase II reactions, including cytochrome P450-mediated oxidations, conjugation, and canalicular transport, after which the resulting metabolites are eliminated via biliary excretion into the small intestine or by renal excretion of water-soluble products [7,26], whereas S molecules undergo metabolic utilization and transformation. Through these coordinated processes, LSECs maintain metabolic and detoxification homeostasis, support hepatic immune tolerance, and preserve the structural integrity of fenestrae [26].

Individual LSECs form small transcellular pores termed fenestrae, approximately 50–200 nm in diameter in mice and ~107 nm in humans [28], covering approximately 6–10% of the endothelial surface [29]. A fenestra-associated cytoskeletal network extends from the cell surface toward the space of Disse and contributes to fenestra structure, number, and dynamics, all of which define LSEC functional activity. Multiple fenestrae within a single LSEC are organized into clusters termed sieve plates, which are interconnected by microtubules and branching cytoskeletal elements [20] and lack an underlying basement membrane [20,25]. These features enable dynamic modulation of fenestra diameter and permit passive, non-endocytic transfer of S, M, and F molecules, their complexes, nanoparticles (<200 nm), and colloids [26]. Larger particles are preferentially cleared by Kupffer cells [30]. However, both clearance pathways may participate in the handling of the same class of molecules, and the relative contribution of each pathway may define the transition from physiological to pathological processing. For example, under physiological conditions, AGEs, as M molecules, are primarily cleared by LSECs via STAB1/2 scavenger receptors [31], whereas AGE accumulation shifts recognition toward RAGE on Kupffer cells, promoting inflammatory responses (Figure 1 right panel) [32]. Fenestrae formation and maintenance are dynamic, actively regulated processes, and their loss results in a less permeable, capillarized LSEC barrier.

Several specific markers of fenestrated LSEC have been identified (Box 1, [9]), including key clearance mannose receptor (Mrc1), type H scavenger receptors and the FcγRIIb receptor for immune complexes. Although these receptors do not function within the fenestrae themselves [25,33], their signaling input is critical for fenestra formation [9], and mice deficient in these receptors exhibit impaired hepatic clearance [31].

Box 1 **Fenestrated LSEC markers.**

**Clearance/scavenger receptors**

**Stab2, Stab1**—Type H scavenger receptors. Uptake of AGE-modified and other electronegative macromolecules;

**Mrc1**—Mannose receptor;

**Fcgr2b (CD32b)**—Low-affinity Fc receptor clearing small immune complexes.

**Endothelial/fenestration machinery**

**Eng (CD105)**—Central TGFβ co-receptor driving ALK1-based signaling;

**Kdr (VEGFR2), Cdh5 (VE-cadherin), Ptprb, Aqp1**—Endothelial vascular integrity;

**Pde2a, Plxnc1**—Markers of differentiated, fenestrated LSEC.

**LSEC identity/secretory markers**

**Oit3, Dnase1L3, Pcxnc1**—Highly selective LSEC markers;

**Ehd3, Igfbp1**—Exosomal/secretory LSEC–parenchymal crosstalk.

**Common external markers overlapping this profile**

**CLEC4G (L-SECtin), LYVE1**—Markers of healthy, fenestrated LSEC aligned with Oit3/Dnase1L3 cluster.

**Genes responsible for functional fenestration**

**Plvap** and **endothelial Slc35a1.** Decreased fenestration and induce steatosis and dyslipidemia and genetically deficient mice [34].

In addition to these physiological clearance and internalization pathways, LSECs, and particularly Kupffer macrophages, express additional scavenger and pattern-recognition receptors (Box 1) that sense PAMP- and DAMP-associated F molecules and activate NF-κB-dependent inflammatory cascades [26,32]. The resulting inflammatory cytokines act on hepatocytes and stellate cells to promote fibrotic and inflammatory responses [26,35], which remodel fenestrated LSECs toward a capillarized phenotype, thereby limiting their clearance and internalization capacity (discussed in Section 4).

## 4. Physiological Regulators of Fenestration–Defenestration

**Figure 2:**
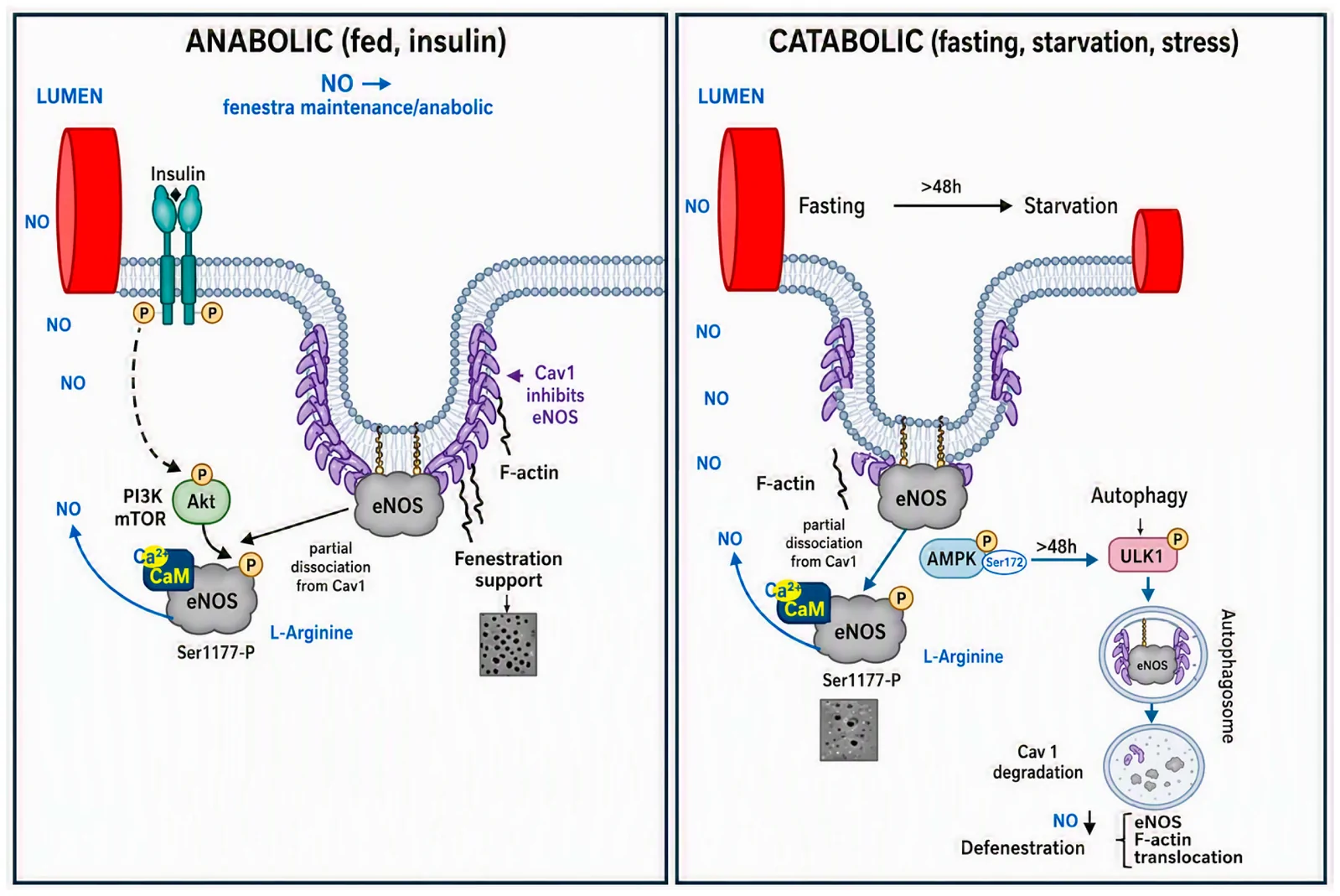
Dynamic and reversible regulation of LSEC fenestration coupled to whole-body metabolic and neuroendocrine status. (**Left** panel): Molecular NO-dependent pathways regulating LSEC fenestration and defenestration during anabolic and catabolic states. Under physiological conditions, eNOS is tonically restrained through its interaction with caveolin-1 (Cav1, violet shape) within caveolar membrane domains. During the anabolic (fed) state, insulin and other anabolic mediators (Figure 3) activate the PI3K–Akt signaling pathway, leading to phosphorylation of eNOS at Ser1177. Concurrent elevations in intracellular Ca^2+^ promote binding of calmodulin (Ca^2+^–CaM) to eNOS and its partial dissociation from Cav1, resulting in full eNOS activation and NO production. NO induces vasodilation and relaxation of the actin cytoskeleton, thereby promoting the formation and maintenance of LSEC fenestrae. Simultaneously, membrane-associated Cav1 redistributes together with F-actin (black wavy lines adjacent to Cav1 dissociated from eNOS) and provides structural support for cytoskeletal remodeling required to establish and stabilize the fenestrated membrane domain. Anabolic hormones (Figure 3) couple nutrient availability to NO production and increased sinusoidal permeability, facilitating efficient exchange and hepatic clearance of circulating metabolites. (**Right** panel): During catabolic conditions (fasting, starvation, or cellular stress), progressive depletion of cellular energy reserves decreases ATP production while increasing intracellular AMP concentrations, thereby elevating the AMP/ATP ratio. AMP-activated protein kinase (AMPK) promotes phosphorylation of eNOS at Ser1177, transiently sustaining NO production to preserve tissue perfusion and metabolic homeostasis despite reduced nutrient availability. However, prolonged nutrient deprivation (>48 h) progressively increases AMPK activation while suppressing mTOR signaling, leading to activation of ULK1 and induction of autophagy. Autophagic degradation of Cav1 disrupts caveolar membrane organization and promotes translocation of Cav1, eNOS, and F-actin, into autophagosomes. The loss of these membrane-associated proteins reorganizes cytoskeleton, reduces NO-dependent maintenance of fenestrae and stabilizes the defenestrated (capillarized) LSEC phenotype. Resumption of dietary intake reverses these catabolic adaptations by restoring anabolic neuroendocrine signaling, thereby re-establishing NO-dependent LSEC fenestration and physiological hepatic clearance.

Physiological LSECs undergo reversible transitions between fenestrated and defenestrated states [36]. These vasostructural and vasodilatory mechanisms regulating fenestration–defenestration cycles rely on the coordinated actions of two principal molecular components: caveolin-1 (Cav1), which governs membrane architecture and cytoskeletal organization [25], and endothelial nitric oxide synthase (eNOS), which regulates nitric oxide (NO)-dependent vasodilatory signaling. Cav1 binds eNOS within caveolar membrane domains, inhibiting NO production and promoting cytoskeletal remodeling associated with the transition toward the defenestrated LSEC phenotype [25,36,37] (Figure 2 left panel, membrane Cav1-eNOS complex [38]). The transition to the fenestrated phenotype is initiated by disruption of the inhibitory Cav1–eNOS interaction through (1) binding of Ca^2+^-bound calmodulin (Ca^2+^–CaM) to eNOS [39], which displaces Cav1, or (2) binding of Ca^2+^–CaM to phosphorylated eNOS (P-eNOS). These interactions generate two NO-generating activation states, Ca^2+^–CaM/eNOS and Ca^2+^–CaM/P-eNOS [40], the latter exhibiting greater catalytic activity and NO production than the former [41]. The resulting NO initiates vasodilation, cytoskeletal remodeling, and LSEC fenestration [42] (Box 2; Figure 2 left panel). Following its dissociation from eNOS, Cav1 assumes a distinct regulatory role in LSEC remodeling. Membrane-associated Cav1 undergoes coordinated redistribution with F-actin during VEGF-mediated cytoskeletal remodeling associated with fenestra formation [43]. In contrast, translocation of Cav1 to autophagosomes promotes its degradation [36,44], thereby stabilizing the defenestrated phenotype (Figure 2, left panel). Thus, through coordinated regulation of NO production and F-actin remodeling, LSEC fenestration is a dynamic, tightly regulated, and reversible process that continuously adapts to extracellular and intracellular signals.

Box 2 **Adaptive regulation of fenestration by scavenging coupled with paracrine pathways.**

Paracrine TGFβ production regulates fenestration markers and scavenger receptors STAB1/2 via the Alk1–TGFβ receptor complex with endoglin, thereby promoting fenestra formation. In contrast, alternative TGFβ receptor complexes involving Alk5 are associated with a defenestrated, capillarized phenotype through activation of SMAD3/SMAD4 transcription factors [9]. This shift can be triggered by clearance of AGE (an M-class foreign-like molecule) via STAB1/2, which requires leptin receptor (LepR) [9] and is accompanied by increased *Kdr* expression, enhancing VEGF responsiveness, sensitivity to neuroendocrine cues, and promotion of fenestration.

### 4.1. Neuroendocrine Regulation of LSEC Fenestration Associated with Postprandial and Other Anabolic States

**Figure 3:**
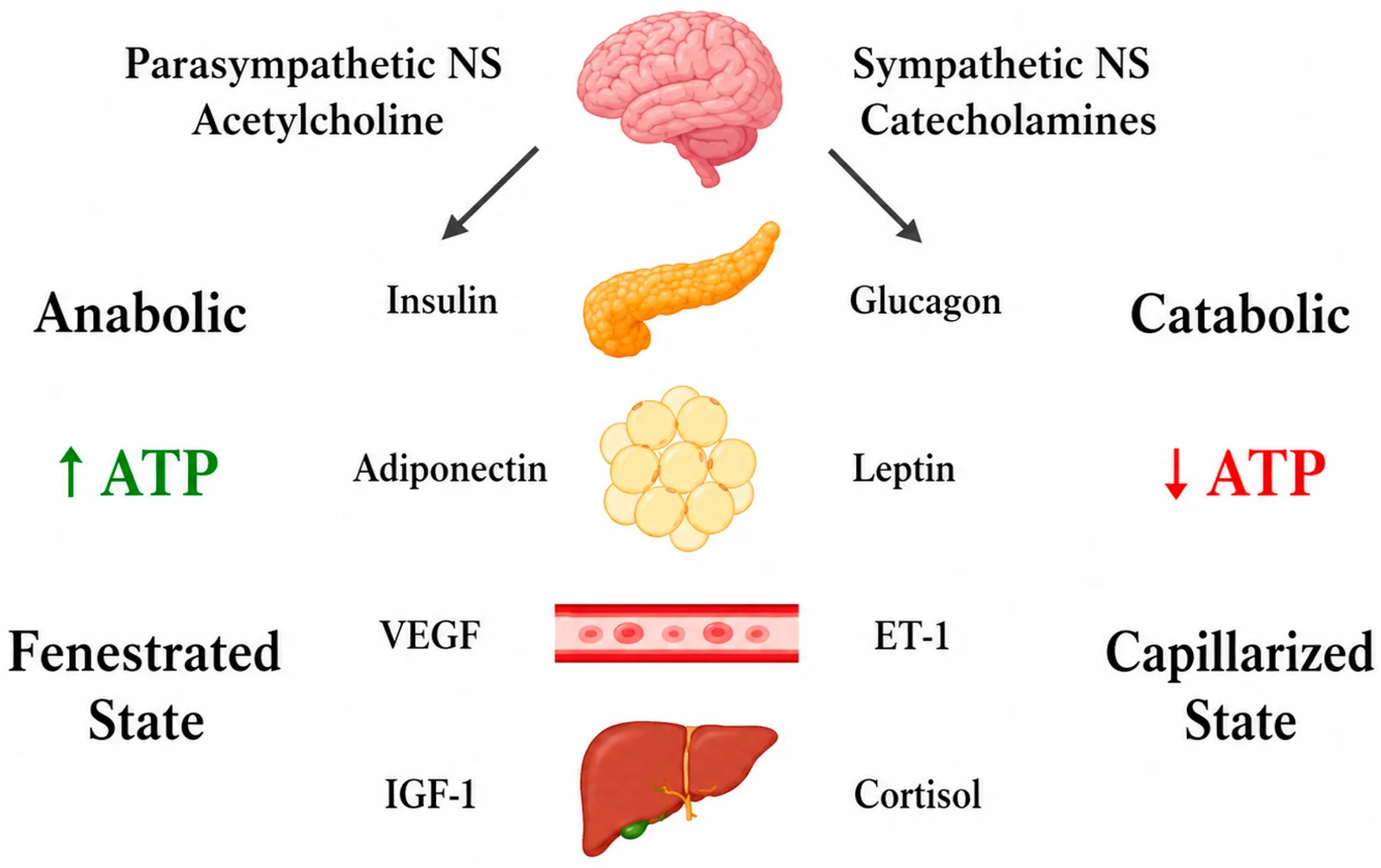
Neuroendocrine and hormonal balance between anabolic and catabolic signaling pathways. This schematic summarizes major regulatory pairs that exert opposing influences on metabolic state and vascular function. Parasympathetic signaling, mediated in part by acetylcholine, is aligned with anabolic regulation, whereas sympathetic signaling, mediated by catecholamines, is aligned with catabolic regulation. The central panel highlights key opposing hormone pairs, including insulin versus glucagon, adiponectin versus leptin, VEGF versus endothelin-1 (ET-1), and IGF-1 versus cortisol. Hepatocyte growth factor (HGF) may also participate in the broader regulatory network that coordinates these pathways to support fenestrations and maintain metabolic homeostasis.

During the postprandial anabolic state, multiple endocrine, metabolic, and neural signals converge on the NO–Cav1 cytoskeletal machinery to preserve the differentiated fenestrated LSEC phenotype. Particularly VEGF and BMP9 [45], together with eNOS signaling are directly implicated in the regulation of NO and maintenance of LSEC fenestrations (Figure 3) [25]. Moreover, classical hormones released during the fed and other anabolic states—including vasoactive intestinal peptide (VIP), insulin, insulin-like growth factor-1 (IGF-1), growth hormone, and the adipokine adiponectin—activate the PI3K–Akt–mTOR anabolic signaling network, stimulating downstream kinases that phosphorylate and activate eNOS, thereby increasing NO production and maintaining vascular homeostasis [46,47,48]. Although their direct effects on LSEC fenestrations remain largely unexplored, these mediators are functionally linked to preservation of the differentiated fenestrated phenotype, which underlies the high hepatic clearance capacity of the liver in fed state [25].

Notably, anabolic states are also characterized by a high ATP-to-AMP ratio, reflecting an energy-replete metabolic state that supports ATP-dependent actin cytoskeletal remodeling and membrane reorganization required for LSEC fenestration [49]. Finally, acetylcholine (ACh), a key mediator of parasympathetic signaling associated with the fed state [50], is widely used as a canonical endothelium-dependent vasodilator [51] to assess LSEC NO/eNOS function in isolated perfused liver [52,53]. Although direct evidence that ACh induces LSEC fenestration or fenestration-associated markers remains limited, selective hepatic parasympathetic denervation increased circulating total cholesterol without affecting body weight or food intake [54]. These findings are consistent with a role for cholinergic signaling in regulating hepatic utilization and clearance of S molecules, although its direct contribution to NO-mediated LSEC fenestration remains to be established.

An additional adaptive mechanism regulating LSEC fenestration in response to increased metabolic waste load involves crosstalk between scavenger receptor-mediated clearance and paracrine signaling pathways activated by F and M molecules, exemplified by electronegative AGEs (Box 2). AGE-mediated feed-forward signaling enhances LSEC scavenging capacity through upregulation of *Stab2* receptors together with increased expression of markers associated with fenestra formation, representing an adaptive response that facilitates hepatic clearance of accumulating metabolic waste [9]. Whether this adaptive response is shared by other endogenous or exogenous waste products remains unknown. Likewise, paracrine signaling through the BMP9–endoglin–ALK1/TGFβ receptor signaling network is essential for maintaining LSEC differentiation, fenestration, and sinusoidal homeostasis [9,49,50]. However, whether this signaling network directly mediates adaptive changes in fenestra formation or fenestra number in response to metabolic waste has not yet been established and warrants future mechanistic investigation.

Thus, fenestrated LSECs facilitate efficient postprandial hepatic clearance of F and M molecules while promoting the rapid transfer and hepatocellular uptake of newly absorbed dietary S molecules. Together, these coordinated responses restore the transient decline in the SFR following nutrient absorption and re-establish systemic metabolic homeostasis.

### 4.2. Neuroendocrine Regulation of LSEC Defenestration Associated with Fasting and Other Catabolic States

In contrast to anabolic states, fasting progressively eliminates the dietary input of F molecules while mobilizing stored S molecules from endogenous energy reserves. Concurrently, the generation of modified self M molecules declines as cellular metabolism shifts from biosynthetic to energy-conserving pathways. These metabolic adaptations are accompanied by a progressive increase in the AMP–ATP ratio, the magnitude and duration of which differ between short-term and prolonged fasting [51]. The elevated AMP–ATP ratio initiates activation of AMP-activated protein kinase (AMPK), which is subsequently reinforced during sustained energy deprivation through phosphorylation and other posttranslational mechanisms that promote autophagy [52]. Concomitant suppression of mTORC1 constitutes an additional essential step for autophagy induction. Autophagy promotes membrane and cytoskeletal remodeling through degradation of Cav1 within autophagosomes and translocation of eNOS and F-actin to the perinuclear compartment, thereby reducing membrane-associated eNOS and limiting NO bioavailability [53]. By 48 h of fasting, LSECs exhibit clear morphological evidence of defenestration, characterized by a reduced fenestral frequency accompanied by compensatory enlargement of the remaining fenestrae [54]. Because fasting and AMPK activation are tightly coupled, we propose that the gradual transition of LSECs from a fenestrated to a defenestrated phenotype serves as a physiological adaptation that prolongs the retention of endogenous S molecules in the circulation, thereby enhancing their availability to extrahepatic tissues during fasting.

Hormone-induced catabolic states are coordinated by glucagon, catecholamines, endothelin-1 (ET-1), serotonin, neuropeptide Y, and the mineralocorticoid aldosterone (Figure 3). Although these mediators act through distinct receptors and signaling pathways, they converge on catabolic metabolic programs that promote energy conservation. Similarly to fasting, the cellular response depends on the balance between receptor-mediated Ca^2+^ signaling and the magnitude and duration of AMPK activation. Transient increases in intracellular Ca^2+^ promote Ca^2+^–CaM binding to eNOS, dissociation of eNOS from Cav1, and NO production, as exemplified by ET-1 acting through endothelial ETB receptors [55]. In contrast, sustained catabolic signaling progressively reinforces AMPK-dependent autophagy, resulting in Cav1 degradation, reduced membrane-associated eNOS, diminished NO bioavailability, and defenestration. In contrast, sustained hormonal stimulation reinforces AMPK-dependent autophagy, leading to Cav1 degradation, reduced membrane-associated eNOS, diminished NO bioavailability, and progressive LSEC defenestration. Chronic catecholamine exposure and aldosterone exemplify this response [36,56]. In particular, aldosterone-induced autophagy has been extensively characterized in LSECs and shown to promote progressive defenestration through the autophagic degradation of Cav1 [36], thereby driving the transition toward the defenestrated phenotype and recapitulating the autophagy-dependent catabolic signaling associated with prolonged fasting.

Other catabolic states, including moderate or exhaustive exercise and catecholamine-induced stress, activate CaM kinases, PKA, and PKG, which phosphorylate eNOS at Ser1177 [57] and converge on the Cav1-dependent eNOS–NO signaling axis. These pathways may transiently preserve endothelial NO production despite systemic catabolic signaling; however, their specific effects on LSEC fenestration–defenestration dynamics have not yet been investigated experimentally.

The rapid onset of stress-induced defenestration and the concurrent presence of high circulating concentrations of S, F, and M molecules distinguish this response from the gradual defenestration associated with fasting, during which F molecules are largely absent and circulating S and M molecule levels progressively decline. We propose that, under these conditions, stress-induced defenestration becomes maladaptive because immune-mediated scavenging of F and M molecules progressively replaces the physiological, immunologically silent clearance performed by LSECs [8], ultimately promoting chronic low-grade inflammation. Thus, fenestrae are not merely morphological features; they dynamically regulate the transport of molecules according to their size, charge, and composition in response to the neuroendocrine signals that coordinate anabolic and catabolic states, thereby determining which molecules reach hepatocytes, which undergo LSEC-mediated clearance, and which remain in the circulation for immune-mediated scavenging.

## 5. Dietary and Nutrient Drivers of Pathological LSCS States

The pathogenesis of LSCS dysfunction and loss of fenestration is fundamentally dependent on the oxidative stress–inflammation axis (Box 3) [58]. Sustained inflammation has emerged as the principal mechanism driving the transition of LSECs from transient, reversible fenestration–defenestration cycles toward persistent capillarization, thereby reducing both the efficacy of hepatic clearance and the utilization of S molecules [8,58]. Clinically and experimentally, chronic inflammation develops progressively in individuals exposed to dietary overload, poor diet quality, or disrupted feeding patterns and is characterized by progression from a single metabolic syndrome to a broader spectrum of metabolic disorders, accelerated aging, and ultimately cancer [4,5]. However, the role of capillarization-associated LSCS dysfunction in perpetuating chronic inflammation and driving the progression of metabolic and age-related diseases remains largely understudied.

To model these inflammatory, capillarized conditions, hepatic clearance efficacy can be treated as effectively approaching zero, such that $\eta_{S}\to 0$, $\eta_{M}\to 0$, and $\eta_{F}\to 0$; consequently, $(1-\eta_{S})\to 1$, $(1-\eta_{M})\to 1$, and $(1-\eta_{F})\to 1$. Under these circumstances, the SFR equation for capillarized LSEC state simplifies to a pre-hepatic clearance state, with the key distinction that, under capillarized inflammatory conditions, reduced hepatic clearance prolongs the residence time of dietary- and metabolically derived S as well as M and F molecules in the circulation. During prolonged circulation, a fraction of S molecules undergoes oxidative, glycative, proteolytic, or other enzymatic modifications, generating an expanding pool of M molecules [8,59], denoted as $X_{m}$. In parallel, $X_{b}$ denotes the fraction of S molecules that are consumed or altered by the microbiome or gut permeability under inflammatory conditions, thereby increasing the pool of F molecules. Thus, the remaining native S molecule pool becomes $\left(S-X\right)$, where $X=X_{b}+X_{m}$, whereas the burdens of F and modified-self M increase to $\left(F+X_{b}\right)$ and $\left(M+X_{m}\right)$, respectively.

$$capillarized\;SFRpost=\frac{S-X}{F+Xb+M+Xm}$$

Consequently, the SFR is progressively reduced, creating a feed-forward cycle in which accumulating M and F molecules further promote retention of circulating molecules, drive molecular dysregulation, and sustain chronic inflammation. With reduced posthepatic clearance, the burden of removing and scavenging F and M molecules steadily shifts to other systems, leading to progressively impaired renal filtration and increased reliance on immune-mediated clearance that perpetuates inflammation [8]. This LSEC capillarization drives the progression of metabolic disease, extends tissue injury and inflammation across an increasingly broad spectrum of organs, and facilitates debilitating processes associated with aging [59]. Recognition of the role of diet in sustaining chronic inflammation and promoting LSEC remodeling provides an additional framework for evaluating the pathogenic potential of foods and dietary patterns.

Box 3 **Major mechanisms of inflammation-dependent LSEC capillarization.**

**Triggers:**—Modified molecules binding to specific receptors (e.g., AGE/RAGE) and modified lipoprotein complexes (e.g., oxLDL, acetyl-LDL) engaging scavenger receptors (SR-A–type) on LSECs or immune cells [35].

- PAMP and DAMP molecules activating TLRs and other pattern-recognition receptors;
- Pro-inflammatory cytokines (e.g., TNF-α, IL-1β) and growth factors (e.g., TGF-β acting via ALK1/ALK5 receptors);
- Reactive oxygen species (ROS) and reactive nitrogen species (RNS).

**Key transcription factors:** NF-κB, HIF-1α, AP-1, SP1, STAT family members

**Mechanisms:**

- ROS/RNS-mediated defenestration.

Inflammatory ROS react with NO to generate RNS, particularly peroxynitrite [60], reducing NO bioavailability [61] required for fenestration. Oxidative and nitrosative damage to cytoskeletal and membrane components disrupts sieve plates. NF-κB-induced iNOS expression further perturbs tightly regulated eNOS-dependent NO signaling and increases RNS production.

- TGF-β-driven shift from ALK1 to ALK5 signaling [9,49,50].

Elevated TGF-β redirects signaling from the pro-fenestration ALK1–endoglin pathway toward ALK5/Smad2/3 and uncouples VEGFR (*Kdr*)-dependent fenestration signaling. This shift promotes extracellular matrix deposition and basement membrane formation, leading to persistent capillarization.

**Outcome:** Impaired reversible fenestration–defenestration cycling, progressive loss of sieve-plate architecture, formation of a continuous basement membrane and capillarized endothelium [8,25].

### 5.1. Nutrient Load, Quality, and Food Processing as Determinants of LSEC Remodeling

**Figure 4:**
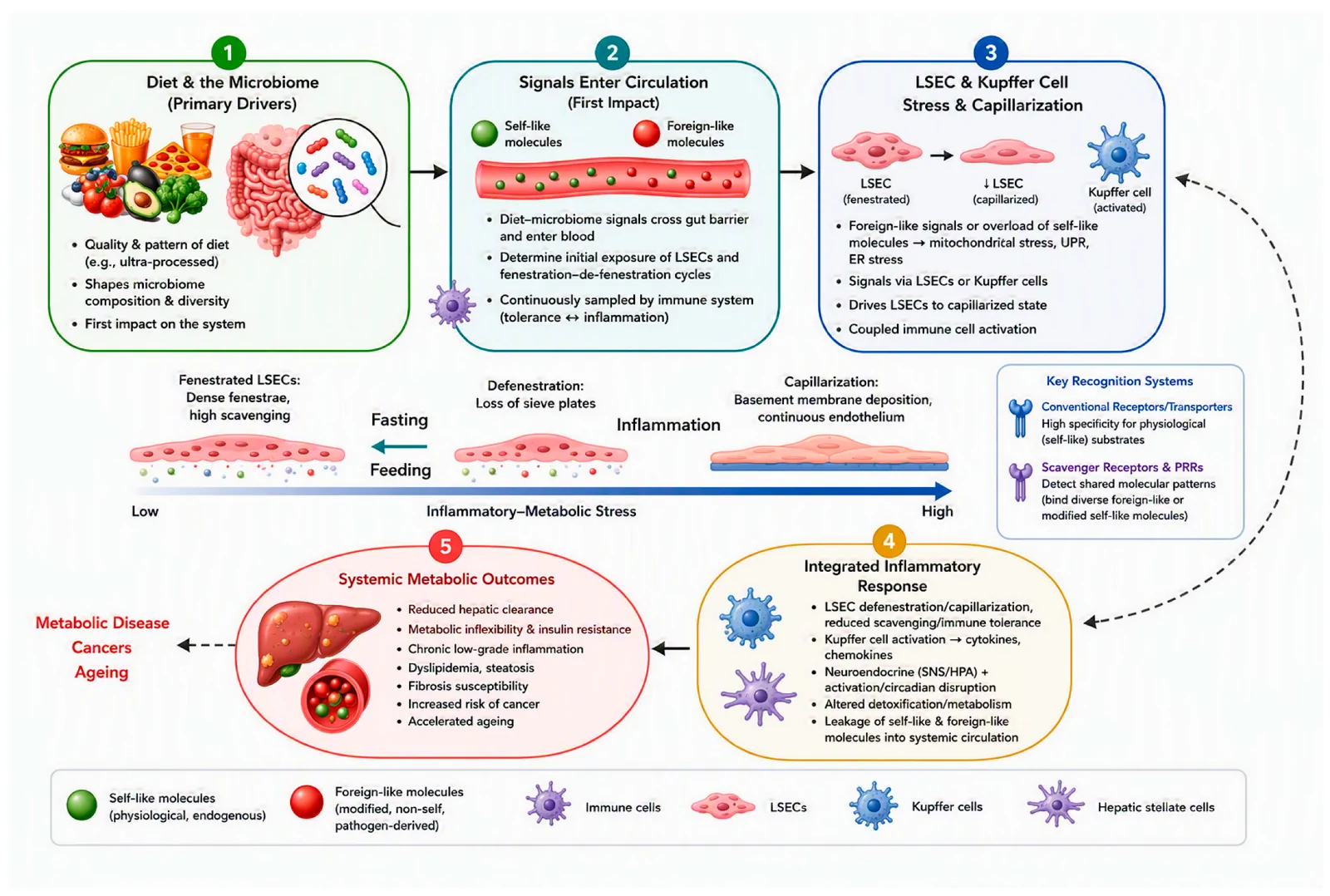
Stage I Dietary input: Diet and eating pattern are the primary upstream drivers. Pathogenic drivers are ultra-processed diets, Western high calories (saturated fat, refined carbohydrates)/high cholesterol and low fiber load, and frequent intake (3–6 meals/day). These dietary factors inducing microbiome remodeling. Pathogenic outcome is dysbiosis, increasing intestinal permeability, and increasing the flux of foreign-like molecules (PAMPs, microbial metabolites, altered bile acids) and self-like molecules postprandial glucose and triglyceride to the liver. Diet–microbiome-derived self and foreign-like signals are sensed by the immune system and, together with neuroendocrine inputs, can shift responses from tolerance toward inflammation. Stage II. Output integration: Within the LSEC, these signals promote a structural trajectory from a healthy fenestrated LSEC (dense fenestrae, high scavenging) through defenestration (loss of sieve plates) under physiological scenario and to chronical sinusoidal capillarization (basement membrane deposition, continuous endothelium, impaired exchange) along an increasing inflammatory–metabolic stress gradient depending either of self-like of foreign-like molecules. Processing of self-like molecule overload can generate intracellular stress in mitochondria characterized by ROS/RNS, unfolded protein response, and ER stress, producing new subset of foreign-like modified molecules, DAMPs, and apoptotic debris, leading to capillarization. Foreign-like molecules can trigger signaling and activate Kupffer and other immune cells, issuing an integrated inflammatory response that amplifies cytokine production, perturbs neuroendocrine signaling (SNS/HPA activation, circadian disruption), and allows self and foreign-like molecules to escape detoxification or metabolism, thereby increasing their systemic levels. Stage III. Metabolic outcome: The resulting systemic metabolic outcomes include reduced hepatic clearance, metabolic inflexibility and insulin resistance, chronic low-grade inflammation, and enhanced fibrogenic signaling, which in turn drive dyslipidemia, steatosis, that are drive metabolic disease and pose risks for cancer. Chronic overload with diet-derived self and foreign-like molecular species accelerate aging.

Since diet provides both nutritional substrates and microorganisms, it shapes gut microbiome composition, gut permeability, and endotoxin levels, including LPS [62]. LSECs are a major clearance system for circulating LPS under physiological conditions, removing LPS–lipoprotein complexes via STAB1/2-mediated endocytosis. However, dysbiosis leads to excessive LPS production and increased gut permeability, progressively shifting LPS signaling toward TLR4-dependent inflammatory pathways, LSEC dysfunction, impairing hepatic clearance, and promoting systemic inflammation and [62]. Fiber-rich, whole-food dietary patterns modulate the microbiota and reduce metabolic endotoxemia [63], although direct evidence of LSEC fenestration rescue or capillarization reversal under such dietary interventions remains limited. Collectively, enrichment of the postprandial circulation with F molecules of microbial origin (Figure 4) constitutes a significant source of chronic inflammation in metabolic diseases and may causatively contribute to LSEC capillarization and diminished hepatic clearance efficacy [64].

Diet quantity cannot be adequately assessed solely by caloric content. Food processing modifies the structure and composition of whole foods by altering the density and bioavailability of macronutrients and by introducing orexigenic and other stimulatory additives, preservatives, and processing-derived compounds [65], thereby increasing consumption, accelerating absorption, and augmenting the portal delivery of both endogenous and foreign-like (F + M) molecular signals. In the United States, ultra-processed foods comprise 57.9% of total energy intake and account for nearly 90% of added sugar calories [66], with more recent data indicating that ultra-processed foods now provide more than half of daily energy intake in US adults [67]. Similar dietary patterns are observed in the United Kingdom, where ultra-processed foods contribute approximately 56% of total energy intake [68], while globally the mean contribution of ultra-processed foods to total energy intake has been estimated at 16–58% [69]. Collectively, these consumption patterns greatly increase chronic exposure to S, M, and F molecules, progressively overwhelming physiological hepatic clearance mechanisms and promoting the accumulation of circulating M and F molecular signals and their inflammatory impact.

Ultra-processed foods [65], typified by the Western-type diet, are characterized by rapid absorption that elicits a pronounced postprandial neuroendocrine response, resulting in combined hyperglycemia, hyperlipidemia, and hyperinsulinemic stress. The addition of refined sugars further amplifies these responses, whereas increasing dietary complexity by adding proteins and lipids—but not fiber—slows absorption and reduces glycemic load and insulin release [70], as well as likely other interdependent anabolic hormones. Although these endocrine signals initially promote anabolic fenestration and hepatic nutrient uptake (Section 4), their effects are progressively outweighed by escalating intracellular and systemic inflammation driven by chronic overconsumption of S molecules. Excessive delivery of fatty acids and glucose overwhelms the metabolic capacity of mitochondria and the endoplasmic reticulum (Box 4), increasing ROS and RNS production and promoting oxidation, glycation, tyrosine nitration, protein misfolding, and other covalent or structural modifications of S molecules, thereby generating pro-inflammatory M molecules [4,59,60].

Box 4 **Major inflammatory mechanisms in response to dietary excess.**

1. Glucose–induced capillarization of LSECs via integrin αv/laminin/phospho-FAK–dependent structural remodeling [71];

2. Mitochondrial oxidative stress [4];

3. Unfolded protein response and ER stress [4].

These inflammatory processes are further amplified by the high energy demands of anabolic nutrient processing, ultimately leading to ATP depletion [4]. Thus, paradoxically, excessive intake of rapidly absorbed nutrients activates two major pathways that promote LSEC capillarization: chronic inflammation and cellular energy depletion [52].

Recent studies demonstrate that, in addition to impairing the clearance of M and F molecules, LSEC capillarization and hepatic inflammation reduce the hepatic uptake of dietary S molecules and lipoproteins [25]. The resulting reduction in hepatic uptake prolongs the circulation of lipoproteins, promoting postprandial hyperlipidemia and ectopic lipid deposition in adipose tissue and other organs [1]. Consistent with this mechanism, genetic disruption of LSEC fenestration recapitulates hypertriglyceridemia and hypercholesterolemia accompanied by the paradoxical development of hepatic steatosis and steatohepatitis despite nutrient abundance in the circulation [34].

Although certain components of ultra-processed foods, particularly fructose, are well-established stimulators of hepatic de novo lipogenesis [72], genetic disruption of LSEC fenestration [34] suggests an additional mechanism. We propose that reduced LSEC fenestration and capillarization impair the hepatocellular delivery of S molecules, creating a state of relative intracellular nutrient insufficiency despite systemic nutrient excess. This perceived nutrient insufficiency may activate compensatory metabolic programs that increase triglyceride, cholesterol, and glucose synthesis [73]. Although direct mechanistic validation of this hypothesis remains to be established, accumulating experimental and clinical evidence supports several of its key components [63,74] (Supplementary Table S1). In particular, chronic systemic inflammation associated with long-term consumption of ultra-processed diets is accompanied by progressive LSEC capillarization, fasting dyslipidemia, and hyperglycemia in metabolic dysfunction-associated steatotic liver disease (MASLD) [63,74].

Within the LSCS framework, chronic inflammation disrupts intestinal barrier integrity, increasing the translocation of microbiome-derived products into the portal and systemic circulation and thereby expanding the pool of pro-inflammatory F molecules. Consequently, the SFR declines, establishing a feed-forward cycle in which chronic inflammation, progressive LSEC capillarization, and impaired intestinal barrier function mutually reinforce one another, further increasing the systemic burden of F and M molecules and exacerbating metabolic dysfunction. Clinically, this self-perpetuating cycle is reflected in the progression from steatosis to MASLD and the broader spectrum of metabolic syndrome-associated disorders [74]. Thus, progressive reduction in the SFR provides a unifying mechanistic framework linking modern dietary patterns, chronic inflammation, LSEC capillarization, and the development of MASLD and related metabolic disorders.

### 5.2. Dietary M and F Molecules: Representative Contributors to LSCS-Dependent Metabolic Dysfunction

The Western diet is a major source of exogenous M molecules, particularly AGE, which are generated when reducing sugars react with proteins or lipids during industrial food processing and high-temperature cooking methods, such as roasting, grilling, and frying, increasing the AGE content of foods by approximately 10- to 100-fold. [75]. Following absorption, AGE-modified circulating proteins, including AGE–albumin, are efficiently internalized by stabilin-1 and stabilin-2 receptors expressed on LSECs, underscoring the central role of the liver sinusoidal scavenging interface in their physiological clearance [12]. At elevated concentrations, however, AGEs binds to RAGE, activating pro-inflammatory signaling pathways [76]. Dietary AGE intake approximately doubles circulating AGE concentrations in diabetic mice [77] and increases circulating AGE levels by up to 30% in humans with diabetes, even when total caloric intake and macronutrient composition are maintained and only cooking methods are modified [78]. Consequently, diet-derived AGE represent a substantial exogenous source of systemic inflammatory stimuli that may promote LSEC capillarization and contribute to the accumulation of circulating AGE and elevated HbA1c observed in aging and diabetes mellitus [9]. However, the impact of LSEC capillarization on the hepatic clearance of AGEs and other diet-derived modified molecules has not yet been systematically investigated.

Synthetic food additives represent a distinct component of the dietary exposome because they are intentionally incorporated to modify sweetness, color, preservation, texture, or oxidative stability. From a pharmacokinetic perspective, these compounds can be broadly classified into hydrophilic F molecules, which are eliminated predominantly through renal excretion, and lipophilic F molecules, which require hepatic uptake and metabolism before elimination [79]. Lipophilic synthetic antioxidants commonly present in processed foods—including butylated hydroxytoluene (BHT), butylated hydroxyanisole (BHA), and tert-butylhydroquinone (TBHQ)—undergo hepatic oxidative and phase II metabolism, including glucuronidation, sulfation, and glutathione conjugation, with their metabolites subsequently excreted in the urine [79]. Under conditions associated with LSEC capillarization, including aging, metabolic disease, and chronic inflammation, impaired sinusoidal transfer of lipophilic F compounds to hepatocytes may reduce the efficiency of hepatic metabolism and clearance, thereby increasing systemic exposure to components of the nutritional exposome.

Understanding how food additives and other dietary constituents regulate LSEC function has been hindered by the lack of stable, reproducible, and physiologically relevant in vitro models. LSECs exhibit marked phenotypic instability ex vivo, with hallmark characteristics—including fenestrations, high endocytic capacity, and specialized scavenger functions—rapidly deteriorating within 48–72 h of culture, thereby limiting the reliability of in vitro dose–response studies of candidate modulators [80]. Consequently, relatively few compounds have been convincingly demonstrated to modulate LSEC function in vivo, and the molecular mechanisms regulating LSEC fenestration and hepatic clearance remain incompletely understood. Most available studies investigate single food additives rather than the complex dietary exposome, limiting our understanding of how nutrients, food additives, and microbiome-derived metabolites collectively regulate hepatic clearance and metabolic homeostasis.

Emerging evidence further suggests that the impact of Western-style diets extends across generations. Maternal consumption of a Western-style diet has been shown to epigenetically program LSEC dysfunction in offspring [81]. In aging populations, diet-induced LSEC dysfunction further synergizes with age-associated cellular senescence to accelerate MASLD progression and fibrosis [82]. Collectively, these findings indicate that inflammation-promoting dietary patterns—including Western-style diets and diets enriched in ultra-processed foods—should be recognized not merely as risk factors for metabolic disease but as major drivers of progressive hepatic clearance dysfunction. Accordingly, dietary intervention represents a critical therapeutic strategy for preserving or restoring LSEC homeostasis and improving outcomes across the spectrum of chronic metabolic diseases.

## 6. Modulation of LSEC Function as a Therapeutic Strategy

The dependence of metabolic disease progression on LSEC defenestration and capillarization presents challenges not only for understanding disease pathogenesis and improving diagnosis but also for therapeutic intervention, because many therapeutic molecules and nanocomplexes enter the same LSCS-mediated clearance pathways as F and M molecules. Moreover, the efficiency of this clearance may itself vary with nutritional status and the metabolic phase of nutrient processing [8]. Moreover, the fenestration–capillarization state of LSECs profoundly influences the pharmacokinetics of therapeutic agents. LSEC capillarization prolongs the circulation time of therapeutic molecules by reducing their hepatic clearance, whereas physiological fenestration promotes their efficient hepatic uptake and clearance [25,58]. Consequently, alterations in LSEC function may modify drug disposition and tissue exposure, potentially influencing therapeutic efficacy and toxicity [83]. Therefore, controlled bidirectional regulation of LSEC fenestration—consisting of transient defenestration followed by restoration of physiological fenestration—may provide an effective strategy to temporarily prolong the circulation time of therapeutic molecules while subsequently promoting the efficient hepatic clearance of accumulated M and F molecules.

### 6.1. Dietary F Molecules as Modulators of LSEC Function

Several dietary molecules have been identified as modulators of LSEC function, although most act indirectly by attenuating inflammatory, oxidative, or metabolic stress pathways rather than directly regulating fenestration (Supplementary Table S1). For example, the free fatty acids palmitic and oleic acid modulate the inflammatory state of cultured LSECs by influencing lipid metabolic pathways while suppressing the expression of pro-inflammatory chemokines [84]. By attenuating inflammatory signaling, these effects may contribute to the preservation of the differentiated LSEC phenotype and oppose inflammatory processes that promote capillarization. Likewise, dietary methylxanthines—including caffeine, theobromine, theophylline, and paraxanthine—modulate LSEC fenestration dynamics; notably, physiologically relevant concentrations of theobromine (8 μg/mL) significantly increase fenestration number, demonstrating that diet-derived xanthines can directly regulate sinusoidal endothelial morphology [85]. Similarly, the active vitamin D metabolite calcitriol [1,25(OH)_2_D_3_] activates VDR to attenuate high glucose-induced capillarization through AMPK-dependent autophagy and suppression of the NLRP3–GSDMD pyroptotic pathway [86], thereby counteracting the detrimental effects of chronic nutrient excess on LSEC function.

Nutrient F molecules exposures differ markedly in both the magnitude and duration of their effects on LSEC morphology, ranging from transient and reversible transitions between fenestrated and defenestrated states to persistent defenestration and capillarization that ultimately compromise hepatic clearance. This distinction among dietary F molecules is critical not only for developing strategies to preserve or restore LSEC function during chronic metabolic stress but also for identifying compounds capable of inducing acute hepatotoxicity, such as coumarins present in *Cinnamomum cassia*, which can cause liver injury in susceptible individuals [87].

### 6.2. Psychoactive Substances as Modulators of LSEC Function

Psychoactive substances represent an important class of F molecules in exposome with the potential to modulate LSEC function and thereby influence hepatic clearance. The widespread therapeutic use and misuse of opioids, analgesics, and other CNS-active compounds, together with the growing global burden of use of addictive substances and ethanol-containing drinks, underscore the importance of understanding their effects on the LSCS [88]. The regulation of LSEC function by CNS-active compounds has been comprehensively reviewed by Szafrańska et al. [25]. Available evidence indicates that several addictive psychoactive substances, including nicotine, ethanol, and cocaine, reduce LSEC fenestration, whereas the psychedelic amphetamine derivative 2,5-dimethoxy-4-iodoamphetamine increases fenestration in both young and aged rodents [25].

As a representative F molecule, ethanol induces morphological changes in LSECs ranging from transient fenestral remodeling to progressive capillarization [89]. Ethanol-induced defenestration has been documented in clinical and experimental studies across rodent and non-human primate (baboon) models, as well as in patients with alcoholic steatohepatitis and perisinusoidal fibrosis. However, the relative contributions of the direct effects of ethanol, the generation of reactive aldehydes and other M molecules, and the indirect systemic effects of ethanol—including CNS dysfunction, dysbiosis, and LPS-mediated inflammation—to LSEC remodeling remain largely unexplored. Unlike MASLD, in which dyslipidemia develops early, LSEC capillarization in alcohol-associated liver disease is accompanied by dyslipidemia primarily in the setting of fibrosis [89], suggesting that chronic ethanol exposure engages additional pathogenic mechanisms beyond impaired fenestration. Furthermore, ethanol potentiates the defenestrating effects of cocaine and acetaminophen, indicating that interactions between psychoactive substances may synergistically impair LSEC function. Although the effects of many other commonly used psychoactive substances—including opioids, amphetamines, and cannabinoids—on LSEC biology remain largely unexplored, their widespread use and potential to alter hepatic metabolism, systemic exposure to xenobiotics, and the pharmacokinetics of co-administered medications warrant further investigation.

## 7. Nanofiber-Based Regulation of LSCS Clearance Function

The epidemic rise in degenerative diseases demands new therapeutic strategies; however, up to 90% of drug candidates entering clinical trials (Phase I–III) ultimately fail to receive regulatory approval [90]. Major contributors to this high attrition rate include insufficient drug exposure, drug-induced toxicity, and hepatic metabolism, all of which reduce therapeutic efficacy [91]. We propose that many of these barriers are fundamentally influenced by the functional state of the LSCS. Consequently, transient and controlled modulation of the fenestration–defenestration balance may provide a strategy to optimize therapeutic exposure while preserving physiological hepatic clearance.

This concept is particularly relevant for nutraceutical- and nanocarrier-based therapies, which encounter multiple sequential clearance barriers, including poor oral bioavailability, LSEC-mediated sequestration of nanosized particles, hepatocyte cytochrome P450 (CYP)-dependent metabolism, and Kupffer cell phagocytosis [92]. Together, these processes limit systemic drug exposure while promoting oxidative stress and the conversion of S and M molecules.

Recent studies have further highlighted the central role of LSECs in nanoparticle clearance. Wang et al. demonstrated the remarkable scavenging capacity of LSECs, which outnumber Kupffer cells by approximately 2.5-fold and efficiently internalize nanosized carriers (~100 nm) while maintaining immune tolerance under inflammatory conditions [93]. Likewise, Jiang et al. used PEGylated liposomal doxorubicin to map its intrahepatic fate, demonstrating that coordinated LSEC–Kupffer cell interactions within the LSCS govern the hepatic uptake, intracellular trafficking, and metabolism of nanocarrier-delivered therapeutics [94]. Current approaches to overcome these barriers have focused primarily on optimizing nanocarrier design and engineering nanoparticle surface properties to improve circulation time, biodistribution, and tissue targeting [92]. Additional pharmacological strategies seek to increase drug exposure through selective inhibition of CYP450-mediated metabolism. However, these approaches are inherently drug-specific and may increase the risk of clinically significant drug–drug interactions [95]. Although these strategies can improve drug delivery, their benefits are generally modest and often require agent-specific optimization, highlighting the need for therapeutic approaches that directly modulate LSCS function.

To address this challenge, recent work has explored amino acid-based supramolecular nanofiber platforms with the capacity to modulate LSCS function [9]. Recent work has focused on amino acid-based nanofibers (AAC2) composed of a coumarin-conjugated dilysine derivative that self-assembles into a highly ordered, positively charged fibers [9]. These nanofibers readily form supramolecular complexes with electronegative proteins, including insulin [96] and insulin-like growth factor-binding protein 4 (IGFBP4) [9]. Importantly, the nanofiber AAC2 itself possesses intrinsic biological activity by functioning as a biologically active F-molecule ligand for the leptin receptor [97], rather than merely serving as a passive delivery vehicle. This dual-function platform combines the biological activities of both the nanofiber carrier and its protein cargo, thereby expanding the therapeutic potential beyond that of conventional nanocarrier systems.

Nanofibers have emerged as highly effective platforms for drug delivery because of their high surface area-to-volume ratio, elongated morphology that can reduce phagocytic clearance, tunable self-assembly and release kinetics, and readily functionalized surfaces that enable multivalent ligand presentation and biomolecular interactions (Supplementary Table S2) [9,96,98]. Treatment of ob/ob mice with AAC2 nanofibers demonstrates programmable regulation of LSEC function [9]. AAC2 alone suppresses the transcriptional program associated with fenestration, whereas its complex with IGFBP4 activates the transcriptional program characteristic of the fenestrated LSEC phenotype. Functionally, the AAC2–IGFBP4 complex increases the expression of the key hepatic clearance receptors stabilin-1, stabilin-2, and FcγRIIb, resulting in a critical functional outcome: prevention of HbA1c accumulation and maintenance of physiological HbA1c levels in the blood of ob/ob mice [9]. Elevated HbA1c is a clinically established marker of chronic protein glycation and serves as an indicator of the sustained burden of glycated proteins in diabetes [78]. These findings suggest that impaired hepatic sinusoidal clearance is a key driver of the progressive accumulation of glycated and other M molecules. Restoration of hepatic sinusoidal clearance by the AAC2–IGFBP4 complex has the potential to reduce the systemic burden of these M molecules, representing a promising therapeutic strategy for obesity, diabetes, and their long-term debilitating complications.

**Figure 5:**
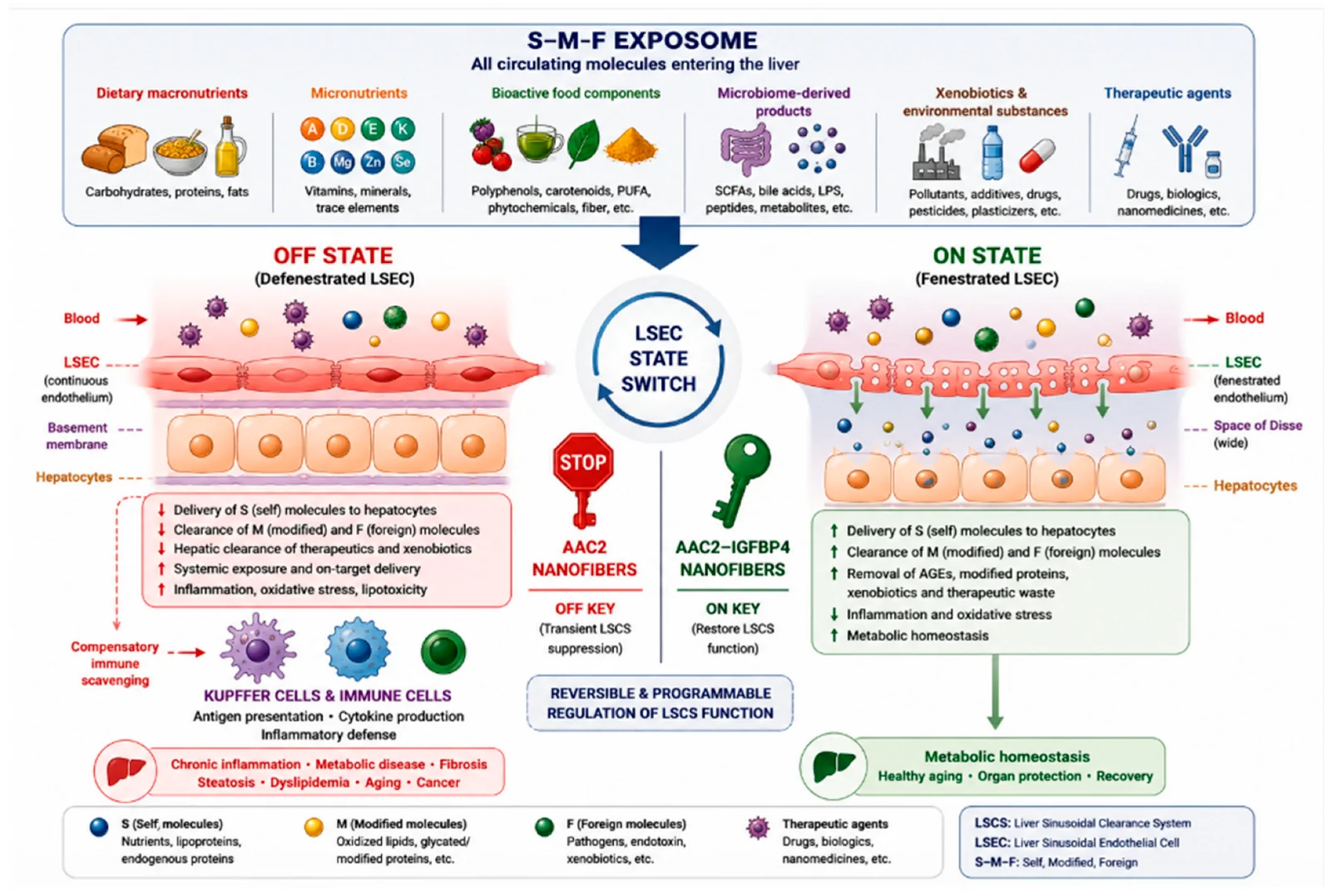
Programmable nanofiber strategies to adapt liver sinusoidal clearance to the composition of the S–M–F exposome. The S–M–F composition of the exposome continuously changes in response to dietary macro- and micronutrients, bioactive food components, microbiome-derived products, xenobiotics, environmental chemicals, and therapeutic agents. These molecular inputs dynamically regulate the reversible ON (green key) and OFF (red key) states of the LSEC. The ON state supports physiological delivery of S molecules to hepatocytes together with efficient clearance of M and F molecules, whereas the OFF state reduces LSCS function and redirects scavenging toward Kupffer cells and other immune cells, promoting inflammatory responses. Programmable supramolecular nanofibers transiently shift LSEC function between these two states: AAC2 induces the OFF state to increase therapeutic exposure, whereas AAC2–IGFBP4 restores the ON state to enhance physiological clearance. We propose that adaptive OFF–ON modulation of the LSCS according to the S–M–F composition of the exposome may improve detoxification, delay metabolic disease, cancer, and aging, and increase the therapeutic index of pharmaceuticals.

Leptin receptor signaling appears to be a fundamental component of AAC2 nanofiber activity. In leptin receptor-deficient db/db mice, the biological effects of free AAC2 and its complexes with either insulin or IGFBP4 were abolished [9,96,97], demonstrating that leptin receptor signaling is required for AAC2-mediated regulation of LSEC function, including modulation of TGFβ-regulated fenestration [9]. Nevertheless, the protein cargo profoundly alters the biological activity of AAC2. Whereas free AAC2 suppresses the expression of fenestration-associated genes, complexation with IGFBP4 activates the transcriptional program characteristic of the fenestrated LSEC phenotype [9]. This activity is specific to IGFBP4, as the AAC2–insulin complex lowers hyperglycemia but does not prevent HbA1c accumulation in ob/ob mice [96]. These findings demonstrate that AAC2 functions as more than a passive nanofiber scaffold; rather, it serves as a programmable therapeutic platform whose biological activity emerges from cooperative interactions between the nanofiber and its associated protein cargo. We propose that, as a foreign F molecule, the AAC2–IGFBP4 complex is recognized by LSECs as a unique regulatory complex that promotes fenestration, potentially through engagement of the endoglin–ALK1–KDR signaling axis [9], although the molecular mechanisms underlying this response remain to be elucidated. The ability to bidirectionally regulate LSEC fenestration—transiently suppressing hepatic clearance to prolong systemic exposure of therapeutic agents or activating hepatic clearance to accelerate the removal of F and M molecules could represent a versatile therapeutic strategy with the potential to enhance the efficacy of both pharmaceuticals and nutraceuticals while mitigating metabolic and neurodegenerative disorders (Figure 5). However, to date, programmable modulation of LSCS by supramolecular nanofibers remains at the preclinical stage, and clinical studies evaluating the AAC2 nanofibers and nutraceutical-based nanotherapeutics provide a promising strategy for overcoming several limitations of conventional therapeutics, particularly rapid hepatic sinusoidal clearance, while enabling programmable regulation of drug exposure and detoxification.

Nevertheless, their clinical translation remains dependent on overcoming several challenges, including scalable manufacturing, batch-to-batch reproducibility under Good Manufacturing Practice (GMP) conditions, and the long-term stability of protein- or nutraceutical-loaded nanocarriers [99]. In addition, the field still lacks robust preclinical evidence demonstrating in vivo efficacy, rigorous in vitro–in vivo correlation (IVIVC), and a detailed mechanistic understanding of how nanocarrier–cargo interactions generate synergistic biological responses [99,100]. Although translating these technologies into clinical practice will require continued advances in nanomaterial design, rigorous mechanistic validation, scalable manufacturing, formulation science, and translational pharmacology, the rational design of multifunctional nanoplatforms capable of modulating hepatic sinusoidal clearance through supramolecular complex formation represents a promising new direction in nanomedicine. By conferring emergent therapeutic functions on proteins, nutraceuticals, and pharmacological modulators, these programmable nanoplatforms have the potential to expand the therapeutic repertoire of existing biomolecules and enable innovative strategies for the prevention and treatment of metabolic, neurodegenerative, and other chronic diseases.

## 8. Conclusions

This review introduces several conceptual advances, including recognition of the LSCS as a central regulator of systemic homeostasis; a novel classification of circulating molecules into self S, modified self M, and foreign F molecules; the self-to-foreign ratio SFR as a systems-level framework for assessing hepatic clearance; integration of feeding-fasting cycles, nutritional, neuroendocrine, inflammatory, and pharmacological regulation of LSEC fenestration; and the concept of programmable bidirectional modulation of hepatic clearance using supramolecular nanofiber therapeutics (Figure 5). Collectively, these advances establish the LSCS as a unifying mechanism linking nutrition, metabolism, immunity, pharmacokinetics, and chronic disease.

Beyond summarizing the current literature, we propose a conceptual framework that helps reconcile several longstanding controversies in the field. First, it places the seemingly conflicting roles of AMPK and eNOS within the physiological continuum from the fed state to fasting and ultimately starvation, suggesting that their reported differences reflect distinct stages of metabolic adaptation rather than contradictory mechanisms. Second, the S–M–F classification and the concept of F molecule recognition provide a new interpretation of the longstanding debate over whether LSEC defenestration is invariably pathological. The accumulated evidence supports our proposal that transient defenestration is a physiological and protective response to foreign pathogens and other F molecules, redirecting their recognition and elimination toward immune cells, whereas persistent capillarization becomes maladaptive by sustaining chronic inflammation and impairing hepatic clearance. Furthermore, the S–M–F framework provides a unifying interpretation of the conflicting mechanisms proposed for the development of dyslipidemia. Rather than viewing altered hepatocyte metabolism and impaired LSEC transport of lipoproteins as competing explanations, our model suggests that these processes are complementary and mechanistically linked through progressive LSEC defenestration, which limits hepatocellular delivery of S molecules lipoprotein complexes while simultaneously impairing the clearance of M and F molecules. This framework also explains why transient, controlled defenestration may be therapeutically advantageous by prolonging systemic exposure and increasing the on-target efficacy of therapeutic agents. Finally, conflicting evidence also exists regarding whether Kupffer cell scavenging compensates for LSCS dysfunction or instead amplifies inflammation. We interpret this apparent contradiction as reflecting a physiological switch from immunologically silent LSCS-mediated clearance to antigen presentation and inflammatory defense. However, immune-mediated scavenging cannot compensate for the loss of LSCS-mediated delivery of S molecules to hepatocytes or prevent the development of steatosis and dyslipidemia associated with chronic capillarization. A comprehensive analysis of hepatic inflammatory mechanisms was beyond the scope of this review and will require dedicated future investigation.

**Figure 6:**
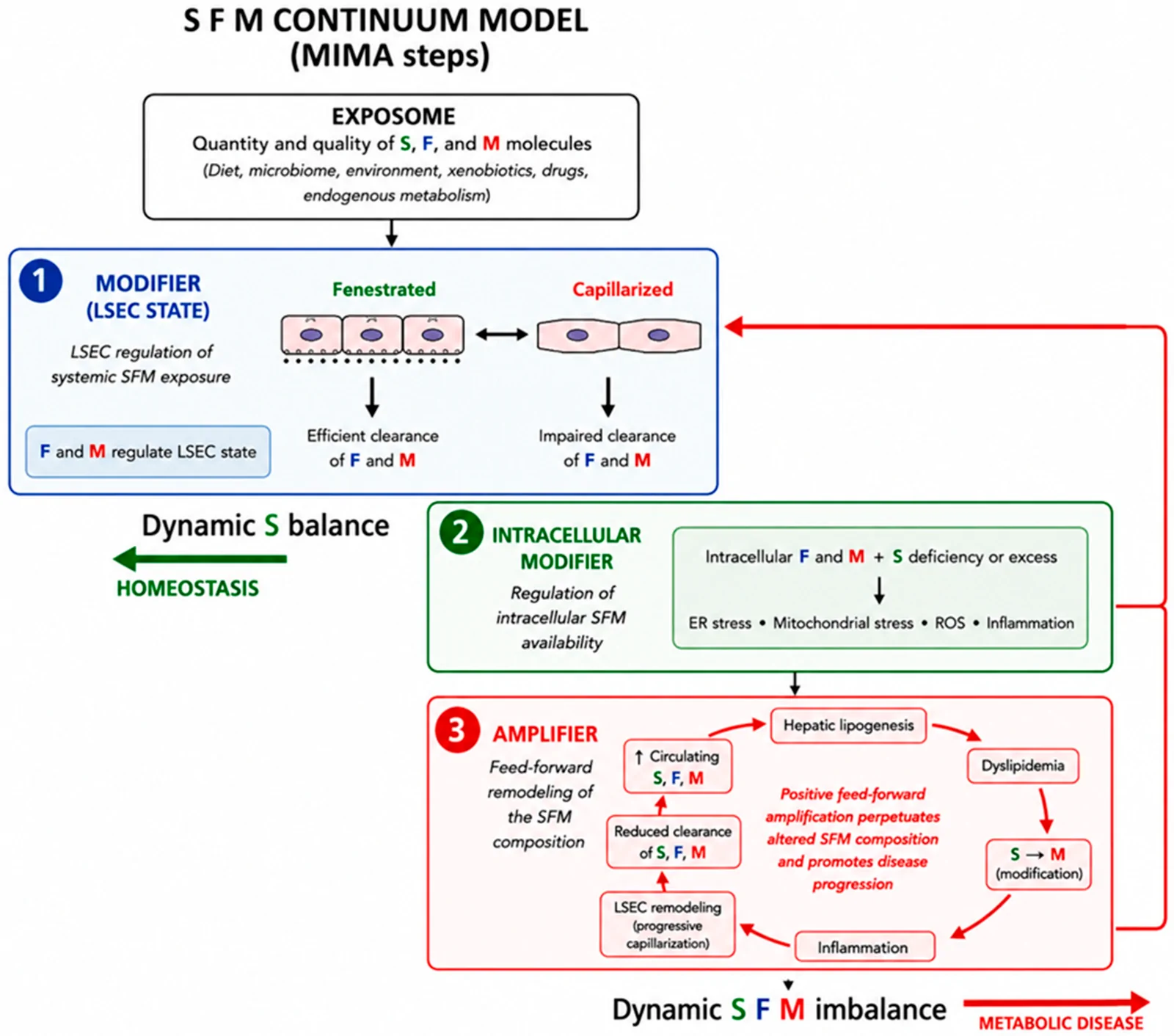
The SFM continuum and the Modifier–Intracellular Modifier–Amplifier (MIMA) model. Dietary quantity, quality, and food processing determine the delivery of S, F, and M molecules to the liver. 1. In the **Modifier** step, F and M molecules regulate inflammation and the LSEC phenotype, thereby controlling hepatic clearance and the circulating SFM composition. 2. In the **Intracellular** Modifier step, intracellular availability of S molecules, together with persistent F and M molecules, determines metabolic homeostasis or, under conditions of chronic deficiency or excess of S molecules, particularly following chronic overeating and consumption of ultra-processed diets, induces ER stress, mitochondrial dysfunction, oxidative stress, and inflammation. 3. In the Amplifier step, adaptive responses to intracellular S deficiency stimulate hepatic lipogenesis, while chronic inflammation promotes the continued conversion of S into M molecules, producing dyslipidemia with progressively increasing circulating S, M, and F molecules. These changes establish feed-forward loops that further impair LSEC function, remodel the SFM composition, and ultimately culminate in chronic metabolic disease. Together, these three sequential processes constitute the proposed MIMA model of the SFM continuum.

We propose that the quantity, quality, and processing of the diet should be viewed through the lens of the delivery and hepatic clearance of S, F, and M molecules, which together define the SFM continuum (Figure 6). The SFM continuum extends the current Diet–Oxidative Stress–Inflammation paradigm by introducing a hierarchical framework in which the biological effects of the exposome are determined through three sequential regulatory steps.

In the first step, the **Modifier**, the quantity and quality of F and M molecules regulate the inflammatory state and, consequently, the LSEC phenotype, thereby determining hepatic clearance and the systemic availability of S, F, and M molecules. By regulating hepatic clearance, the LSEC state establishes the circulating SFM composition that is delivered to peripheral tissues.

In the second step, the **Intracellular Modifier**, F and M molecules are either efficiently cleared by fenestrated LSECs or persist in the circulation when LSECs become capillarized, thereby altering intracellular exposure. The resulting intracellular availability of S molecules determines whether they support metabolic homeostasis or, under conditions of chronic deficiency or excess, induce ER stress, mitochondrial dysfunction, oxidative stress, and inflammation, further promoting LSEC capillarization. Ultra-processed diets promote the rapid absorption of S molecule, whereas LSEC capillarization reduces their availability to hepatic cells, thereby triggering compensatory de novo synthesis. 

In the third step, the **Amplifier**, adaptive responses including hepatic lipogenesis, together with chronic inflammation, promote the continued conversion of S into M molecules, resulting in dyslipidemia and a progressive shift in the circulating SFM composition toward increased S, M, and F levels. These changes establish feed-forward loops that further amplify inflammation, impair LSEC function, reduce hepatic clearance, and perpetuate remodeling of the SFM continuum, ultimately culminating in chronic metabolic disease.

Together, these three sequential regulatory processes constitute the Modifier–Intracellular Modifier–Amplifier (MIMA) model, which defines the proposed SFM continuum.

These LSEC-dependent molecular dynamics provide a unifying framework for understanding both physiological metabolic homeostasis and the paradoxical coexistence of elevated circulating metabolites with excessive hepatic lipogenesis characteristic of steatotic liver disease and related metabolic disorders. The regulatory and clearance biomarkers *Eng*, *Stab2*, and *Fcgr2b* are promising candidates for clinical studies assessing metabolic status and early therapeutic responses. Combined with the differentiated, fenestrated LSEC markers soluble *Oit3* and tissue-specific *Dnase1L3*, they may provide a biomarker panel for the diagnostic assessment of LSEC fenestration, a fundamental determinant of hepatic clearance function. 

From a therapeutic perspective, pharmacological agents, as F molecules, are subject to the same hepatic recognition and clearance mechanisms, creating both a challenge and an opportunity for precision medicine. Recent advances in supramolecular nanotechnology demonstrate that hepatic sinusoidal clearance can be bidirectionally regulated [9], enabling either prolonged systemic exposure to therapeutic agents or accelerated removal of metabolically harmful molecules. Clinical translation of therapies directly targeting LSEC fenestration and LSCS clearance remains at an early stage. To date, registered clinical trials have primarily evaluated agents that indirectly improve hepatic endothelial function, intrahepatic vascular resistance, or metabolic liver disease rather than therapies specifically designed to regulate LSEC fenestration or clearance activity. Statins, particularly simvastatin (ClinicalTrials.gov ID NCT03654053), represent the most clinically advanced endothelial-directed strategy and have been evaluated for their ability to increase hepatic NO bioavailability and reduce portal pressure in cirrhosis. Emerging evidence further indicates that semaglutide exerts weight-loss-independent hepatoprotective effects through GLP-1 receptors expressed by LSECs, as demonstrated in endothelial-specific Glp1r^Tie2-/-^ mice, identifying LSECs as a previously unrecognized mediator of an established metabolic therapy [101]. Direct restoration of LSEC fenestration remains preclinical and includes soluble guanylate cyclase stimulation with riociguat [102], miR-325-3p spherical nucleic acids [103], SO_2_-releasing nanomotors [104], and spermidine-based carbon quantum dots [105]. These approaches pharmacologically modulate NO signaling, cytoskeletal remodeling, and mechanotransduction pathways to restore the fenestrated phenotype. However, none have yet demonstrated the cargo-dependent, bidirectional OFF–ON regulation of LSCS activity achieved by the AAC2 and AAC2–IGFBP4 nanofiber platform. Importantly, programmable supramolecular nanoplatforms can generate emergent biological functions that neither the carrier nor its cargo possesses individually, establishing an entirely new class of therapeutics. The ability to intentionally reprogram hepatic sinusoidal clearance therefore represents a new therapeutic paradigm with the potential to expand the biomedical applications of proteins, nutraceuticals, and pharmacological agents and to transform the prevention and treatment of metabolic, neurodegenerative, and age-related diseases.

## Figures and Tables

**Table 1:** Examples of modified M and foreign F cleared by the LSCS, the receptors involved, and the supporting studies. ECM, extracellular matrix.

Category	Compounds Removed Via LSCS	Primary Receptor(s)	Studies
Modified Proteins	AGEs	Stabilin-1 and -2	[9]
	Oxidized albumin	Stabilin-1 and -2	[12]
Modified Lipid	Oxidized low-density lipoproteins (LDL)	Stabilin-1	[13]
	Acetylated LDL	HARE/Stabilin-2	[14]
ECM Components	Denatured collagen	MR, CD206	[15]
	Pro-collagen peptide	Stabilin-2	[8]
	Hyaluronan	Stabilin-2	[14]
	Chondroitin sulfate	Stabilin-1	[16]
Pathogen-derived	Lipopolysaccharides	Stabilin-1 and -2 or TLR4	[17]

## Data Availability

No new data were created or analyzed in this study. Data sharing is not applicable to this article.

## Supplementary Materials

The following supporting information can be downloaded at: https://www.mdpi.com/article/10.3390/biomedicines14081834/s1, Table S1. Comparative effects of dietary interventions and nutrients on LSEC structure and function; Table S2. Comparative Overview of Supramolecular Nanofiber Systems for Protein Delivery.

## Abbreviations

The following abbreviations are used in this manuscript:

AGE	Advanced Glycation End Product(s)
AMPK	AMP-Activated Protein Kinase
ATP	Adenosine Triphosphate
Cav1	Caveolin-1
DAMP	Damage-Associated Molecular Pattern
ECM	Extracellular Matrix
eNOS	Endothelial Nitric Oxide Synthase
ER	Endoplasmic Reticulum
ET-1	Endothelin-1
F	Foreign Molecule(s)
FcγRIIb	Fc Gamma Receptor IIb
GPCR	G Protein-Coupled Receptor
HbA1c	Glycated Hemoglobin A1c
HIF	Hypoxia-Inducible Factor
IGFBP4	Insulin-Like Growth Factor-Binding Protein 4
IL	Interleukin
KC	Kupffer Cell(s)
LPS	Lipopolysaccharide
LSCS	Liver Sinusoidal Clearance System
LSEC	Liver Sinusoidal Endothelial Cell(s)
MASLD	Metabolic Dysfunction-Associated Steatotic Liver Disease
M	Modified Self-Molecule(s)
MAMP	Microbe-Associated Molecular Pattern
MIND	Mediterranean–DASH Intervention for Neurodegenerative Delay
mTORC1	Mechanistic Target of Rapamycin Complex 1
NADPH	Nicotinamide Adenine Dinucleotide Phosphate (Reduced Form)
NF-κB	Nuclear Factor Kappa B
NO	Nitric Oxide
PAMP	Pathogen-Associated Molecular Pattern
PKC	Protein Kinase C
PRR	Pattern Recognition Receptor
ROS	Reactive Oxygen Species
S	Self-Molecule(s)
scRNA-seq	Single-Cell RNA Sequencing
SFR	Self-to-Foreign Ratio
Stab1	Stabilin-1
Stab2	Stabilin-2
TEM	Transmission Electron Microscopy
TLR	Toll-Like Receptor
VEGF	Vascular Endothelial Growth Factor
