# Supplementary material for: Dietary, Nutrient, and Supramolecular Nanofiber Modulation of the Liver Sinusoidal Clearance System in Metabolic Diseases and Aging
Source: Biomedicines. 2026 Aug 14;14(8):1834. doi: 10.3390/biomedicines14081834 (PMC13509552; doi:10.3390/biomedicines14081834)
Supplement: Supplementary file 1 [file biomedicines-14-01834-s001.zip › biomedicines-4448776-supplementary.pdf]

Supplementary Materials.

Content

Supplementary Table S1
Supplementary Table S2
References

Supplementary Table S1. Comparative effects of dietary interventions and nutrients on LSEC structure and function.

Dietary intervention / nutrient	Molecular target(s)	Experimental model	Principal findings	Clinical evidence	Studies
Coffee/cocoa xanthene's (caffeine, theobromine, theophylline, paraxanthine)	cAMP signaling, cytoskeletal remodeling, fenestration dynamics	Primary rat LSECs (in vitro)	Theobromine at physiologic concentrations (~8 µg/mL) increased fenestration number; higher xanthine exposure altered fenestration morphology and porosity	Preclinical only; no direct LSEC-specific human data	[1]
Vitamin D metabolite $1,25(OH)_2D_3$	VDR, AMPK activation, inhibition of NLRP3 inflammasome and GSDMD-mediated pyroptosis	High-glucose-induced LSEC dysfunction model (in vitro)	Restored autophagy, reversed capillarization, and preserved fenestration structure and endothelial function under metabolic stress	Preclinical only; indirect clinical relevance in metabolic disease	[2]
Free fatty acids (e.g., palmitic, oleic acid)	Lipid metabolic pathways, chemokine signaling (CCL2, CXCL10, CXCL16), inflammatory signaling	Primary LSECs and LSEC cell lines (in vitro)	Modulated lipid metabolism and reduced pro-inflammatory chemokine expression; effects depend on fatty acid composition and metabolic context	Preclinical evidence; indirect clinical relevance in NAFLD but no LSEC-specific human data	[3]
High-fat / Western-style diet<br>Controversial; preclinical evidence only	Oxidative stress pathways, inflammatory signaling (NF-κB), endothelial dysfunction pathways<br>SEMA3A–NRP1–LIMK1–cofilin signaling and reduced LSEC fenestration<br>VCAM1 driven inflammation<br>HFD in C57Bl/6 male mice	Rodent models of diet-induced obesity (DIO) and NAFLD<br>SEMA3A-/- mice and DIO<br>EC specific VCAM1-/- mice<br>Choline-deficient high-fat diet (CD-HFD) to induce NASH	Induce metabolic and inflammatory LSEC dysfunction; effects on fenestrations are model-dependent. Fenestrae may remain preserved during HFD-induced steatosis, whereas reduced porosity and capillarization occur in selected advanced or choline-deficient MASH models.<br>Increased fenestration-related porosity and reduced hepatic lipid accumulation Increased fenestration reduced fibrosis and inflammation<br>Developed obesity, NAFLD, and a pronounced pro-inflammatory LSEC phenotype without detectable changes in fenestration or porosity. LSEC morphology was evaluated by SEM and AFM.	Strong pre clinical association with metabolic disease, but no direct LSEC-targeted	[4];[5]<br>[6]<br>[7]<br>[8]<br>Findings remain controversial because fenestration was assessed primarily by SEM and AFM morphology without stereological quantification of porosity or fenestration frequency

Supplementary Table S2. Comparative Overview of Supramolecular Nanofiber Systems for Protein Delivery

Supramolecular nanofiber system	Physicochemical characteristics	Targeting strategy	Therapeutic payload	Advantages	Disadvantages / limitations	Study
Peptide amphiphile nanofibers	Self-assembling, high aspect ratio structures with tunable rigidity, surface chemistry, and nanoscale organization	Intrinsic presentation of bioactive ligands; matrix-mimetic interactions	Peptides, growth factors, proteins	Biocompatible, modular, and capable of presenting multiple bioactive signals	Potential batch-to-batch variability, stability challenges, and scale-up limitations	[9]
Hybrid nanofiber-nanoparticle systems	Increased loading capacity with combined porosity, mechanical strength, and structural versatility	Surface functionalization and/or encapsulation-based targeting	Drugs, peptides, proteins, genes	Improved encapsulation efficiency and multifunctionality	More complex fabrication, characterization, and quality control	[10]
Electrospun nanofibers incorporating bioactive molecules	High surface area, porous architecture, tunable fiber diameter, and interconnected fibrous networks	Passive local delivery or ligand-functionalized targeting	Growth factors, proteins, small molecules	Cost-effective, scalable, high loading capacity, and tunable release profiles	Primarily suited for local delivery; limited systemic targeting and in vivo biodistribution	[11]
AAC2-based supramolecular nanofibers	Self-assembling coumarin-conjugated dilysine nanofibers that form reversible supramolecular protein complexes	Intrinsic LepR activation; cargo-dependent and cargo independent modulation of LSEC phenotype and LSCS function	Therapeutic proteins (e.g., insulin, IGFBP4)	Modular supra-molecular platform enabling program-mable bidirectional regulation of LSCS activity, reversible modulation of hepatic clearance, prolonged protein bioactivity, and biological synergy between the nano-fiber and its protein cargo	Preclinical stage; manufacturing scalability, long-term safety, and clinical translation remain to be established	[12-14]

References

1. Mao, H., Szafranska K., Kruse L., et al.: Effect of caffeine and other xanthines on liver sinusoidal endothelial cell ultrastructure. Sci Rep 13 (1), 13390 (2023).
2. Jiang, P., Liu Y., Liu J., et al.: Vitamin D Attenuates Hepatic Sinusoidal Capillarization in Type 2 Diabetes Mellitus- Metabolic Dysfunction-Associated Fatty Liver Disease via Dual Autophagy Activation and Pyroptosis Suppression in Liver Sinusoidal Endothelial Cells. Biomedicines 13 (6), (2025).
3. McMahan, R.H., Porsche C.E., Edwards M.G., et al.: Free Fatty Acids Differentially Downregulate Chemokines in Liver Sinusoidal Endothelial Cells: Insights into Non-Alcoholic Fatty Liver Disease. PLoS One 11 (7), e0159217 (2016).
4. Pasarin, M., La Mura V., Gracia-Sancho J., et al.: Sinusoidal endothelial dysfunction precedes inflammation and fibrosis in a model of NAFLD. PLoS One 7 (4), e32785 (2012).

5. Miyao, M., Kotani H., Ishida T., et al.: Pivotal role of liver sinusoidal endothelial cells in NAFLD/NASH progression. Lab Invest 95 (10), 1130–1144 (2015).
6. Eberhard, D., Balkenhol S., Koster A., et al.: Semaphorin-3A regulates liver sinusoidal endothelial cell porosity and promotes hepatic steatosis. Nat Cardiovasc Res 3 (6), 734–753 (2024).
7. Guo, Q., Furuta K., Islam S., et al.: Liver sinusoidal endothelial cell expressed vascular cell adhesion molecule 1 promotes liver fibrosis. Front Immunol 13 983255 (2022).
8. Kus, E., Kaczara P., Czyzynska-Cichon I., et al.: LSEC Fenestrae Are Preserved Despite Pro-inflammatory Phenotype of Liver Sinusoidal Endothelial Cells in Mice on High Fat Diet. Front Physiol 10 6 (2019).
9. Webber, M.J., Berns E.J., Stupp S.I.: Supramolecular Nanofibers of Peptide Amphiphiles for Medicine. Isr J Chem 53 (8), 530–554 (2013).
10. Vargas-Molinero, H.Y., Serrano-Medina A., Palomino-Vizcaino K., et al.: Hybrid Systems of Nanofibers and Polymeric Nanoparticles for Biological Application and Delivery Systems. Micromachines (Basel) 14 (1), (2023).
11. Hiwrale, A., Bharati S., Pingale P., et al.: Nanofibers: A current era in drug delivery system. Heliyon 9 (9), e18917 (2023).
12. Lee, A., Castelli S.L., Sellers P., et al.: Bidirectional regulation of liver sinusoidal clearance by amino acid nanofibers and IGFBP4 complex: effects on HbA1c. J Nanobiotechnology 24 (1), 85 (2025).
13. Lee, A., Mason M.L., Lin T., et al.: Amino Acid Nanofibers Improve Glycemia and Confer Cognitive Therapeutic Efficacy to Bound Insulin. Pharmaceutics 14 (1), (2021).
14. Lee, A., Sun Y., Lin T., et al.: Amino acid-based compound activates atypical PKC and leptin receptor pathways to improve glycemia and anxiety like behavior in diabetic mice. Biomaterials 239 119839 (2020).
